# The Combination of Propofol and Ketamine Does Not Enhance Clinical Responses to Electroconvulsive Therapy in Major Depression—The Results From the KEOpS Study

**DOI:** 10.3389/fphar.2020.562137

**Published:** 2020-09-15

**Authors:** Jerome Brunelin, Sylvain Iceta, Marion Plaze, Raphaël Gaillard, Louis Simon, Marie-Françoise Suaud-Chagny, Filipe Galvao, Emmanuel Poulet

**Affiliations:** ^1^ CH Le Vinatier, Bron, France; ^2^ INSERM, U1028, CNRS, UMR5292, Lyon Neuroscience Research Center, PSYR2 Team, Université de Lyon, Lyon, France; ^3^ Obesity Research Center, Quebec Heart and Lung Institute (IUCPQ), Québec, QC, Canada; ^4^ School of Nutrition, Laval University, Québec, QC, Canada; ^5^ GHU Paris Psychiatrie et Neurosciences, Hôpital Sainte Anne, Paris, France; ^6^ Service de psychiatrie des urgences, Hôpital Edouard Herriot, Hospices Civils de Lyon, Lyon, France

**Keywords:** ketamine, electroconvulsive therapy, major depressive episode, propofol, bipolar

## Abstract

**Objective:**

We investigated the clinical effects of the combination of ketamine and propofol as anesthetic agents during electroconvulsive therapy (ECT) in patients with uni- or bipolar major depressive episodes. We hypothesized that ketamine may confer short- and long- term advantages in improving depressive symptoms at the early stages of ECT.

**Methods:**

In a randomized placebo-controlled trial, remission rates after 4 and 8 weeks of ECT were compared between patients who were randomly allocated to receive either the combination of ketamine (0.5 mg/kg) + propofol (n= 11) or placebo + propofol (n = 16). Depressive symptoms were assessed weekly using the Montgomery–Åsberg Depression Rating Scale (MADRS); ECT sessions were administered twice per week for a maximum of 8 weeks (16 sessions).

**Results:**

After 4 weeks, we observed significantly fewer remitters (MADRS score < 10) in the ketamine + propofol group (0/11; 0%) than in the placebo + propofol group (5/16; 31%; χ^2^ = 4.22; p = 0.040). No significant difference was observed between the two groups regarding the number of patients who achieved remission weekly throughout the study period (Chi² = 3.588; p = 0.058). The mean duration of seizures was significantly shorter in the ketamine + propofol group than in the placebo + propofol group.

**Conclusions:**

The results from the current study corroborated results from previously published studies and did not support the use of the combination of ketamine + propofol as an anesthetic agent for ECT in patients with major depressive episodes in clinical settings.

## Introduction

Depression is one of the most disabling psychiatric conditions, and it had a very high worldwide prevalence (WHO, 2017). In the case of severe and/or treatment-resistant major depressive episodes (MDEs), electroconvulsive therapy (ECT) is commonly proposed as a therapeutic solution. In such cases, ECT has been reported to be a highly effective intervention with a response rate estimated at more than 74% (Bahji et al., 2019) and a remission rate above 50% (Dierckx et al., 2012). However, a range of factors, including a delay of the antidepressive effect and disabling cognitive side effects, limit the use of ECT (Waite and Easton, 2013). An important approach to improve the therapeutic effect of ECT, decrease suicidality, limit the side effects and thus decrease stigma associated with the use of ECT for MDE may be to add psychotropic medications or anesthetic agents during ECT.

Among the candidates, ketamine could be a good choice for several reasons. First, low doses of ketamine are increasingly used due to its antidepressive properties, and several lines of research have revealed its efficacy in the acute treatment of severe major depressive episode (McGirr et al., 2015). Second, due to its pharmacological properties, ketamine alone or in combination with other drugs can also be used as an anesthetic agent during ECT. Some studies that used ketamine during ECT have shown promising results and concluded that ketamine can increase or accelerate the clinical response to ECT (Loo et al., 2012; Wang et al., 2012; Zhong et al., 2016) and decrease cognitive side effects associated with ECT (Kranaster et al., 2011; Shams Alizadeh et al., 2015; Zhong et al., 2016). However, further randomized controlled studies failed to demonstrate the superiority of ketamine (Abdallah et al., 2012; Anderson et al., 2017; Fernie et al., 2017; Zhang et al., 2018). In a meta-analysis of randomized controlled trials, McGirr and colleagues (2017) concluded that the use of ketamine in the ECT setting was not associated with greater improvements in depressive symptoms, higher rates of clinical response, higher rates of remission, or procognitive effects. However, many variations in ECT methodology, including electrode placement (e.g., bitemporal or right unilateral), the method of titration, the use of ketamine in combination with other anesthetic agents or alone, the number and nature of other treatments, the dose and method of administration of ketamine, the depression rating scales used (and definition of response), and the severity of depression at baseline, limit the generalizability of the conclusions that can be drawn from these previously published works and claimed for further studies. Moreover, recent meta-analyses (Ren et al., 2018; Zheng et al., 2019) concluded that although ketamine alone did not appear to improve the efficacy of ECT, ketamine in combination with other anesthetic agents may confer short-term advantages in improving depressive symptoms at the early stages of ECT.

Here, we present the results from the Ketamine for ECT: Optimization Strategy (KEOpS) study, a randomized double blind placebo-controlled study where we compared the remission rate after 8 and after 16 ECT sessions between patients who received the combination of low dose ketamine + propofol and patients who received propofol combined with placebo. We hypothesized that a higher number of patients would achieve remission after eight sessions in the ketamine + propofol group than in the placebo + propofol group. As a secondary objective, we compared the number of remitters after a maximum of 16 ECT sessions, the number of responders and the changes in depressive scores throughout the study period between the two groups.

## Material and Methods

### Sample

**Figure 1:**
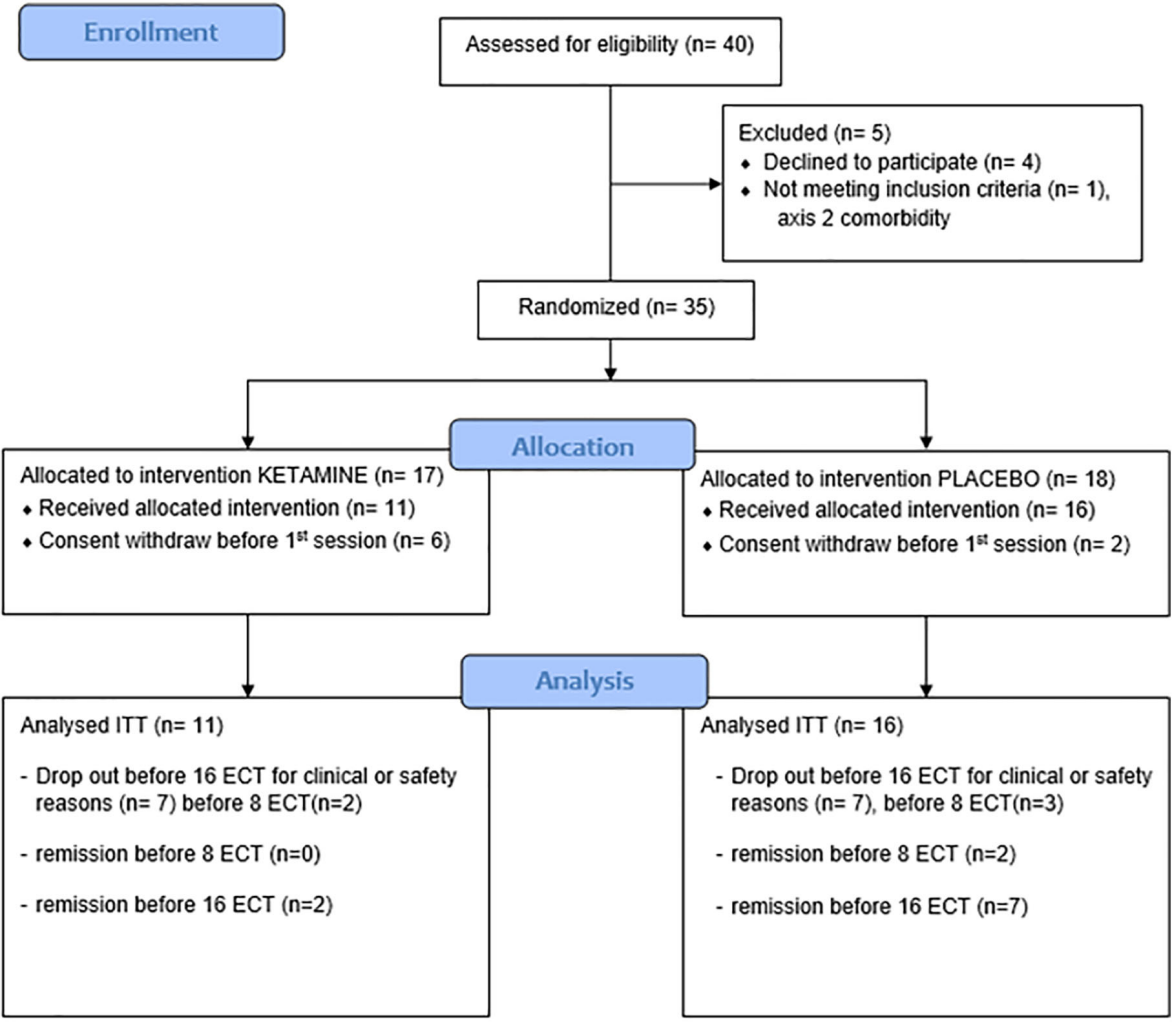
Consort flow diagram of participants.

In a double blind randomized placebo-controlled parallel design study, 40 patients with unipolar or bipolar MDE according to the DSM IV TR criteria were assessed for eligibility between March 2012 and May 2015. In this two-arm parallel study, patients were randomized to receive either ketamine or propofol. The randomization list (1:1 allocation ratio by block of 4) was generated and managed by the sponsor of the study without any intervention of investigators. The study was approved by an ethics committee (CPP sud Est 6, France) and registered in a database for interventional clinical trials (AFSSAPS-EudraCT number 2011-04717-16). All participants signed a written informed consent after a detailed description of the study. Patients had to be free of any other psychiatric or somatic condition and present with a severe MDE (defined by a Montgomery–Åsberg Depression Rating Scale - MADRS score >20) to be eligible for this study. Among the 40 patients screened for eligibility, 13 patients were excluded from the analysis: eight patients were excluded because of withdrawn consent, one because of axis II comorbidity (cluster B) and four declined to participate. The final analyzed sample included 27 patients (see **Figure 1**).

### ECT Parameters

ECT sessions were administered twice per week using a Spectrum 5000Q (Mecta Corporation, Tualatin, OR, USA). Patients received between 8 and 16 ECT sessions until remission. Remission was defined by a MADRS score < 10 (Zimmerman et al., 2004). The duration of seizures (clinical and EEG) was monitored throughout the study period. The severity of depressive symptoms was measured each week with the MADRS. All patients were treated with either right unilateral (RUL) or bitemporal (BT) stimulation according to the patients’ cognitive complaints and ECT practitioner decisions. The seizure threshold (ST) was determined according to a titration method (Poulet et al., 2003). ST was defined as the minimal electrical stimulus charge eliciting a generalized seizure lasting at least 20 s as measured with EEG. ST was individually determined according to a titration schedule during the first session, and treatment was administered at six times the ST for RUL stimulation and at 2.5 times the ST for BT stimulation. The pulse duration was ultrabrief (0.3 ms) during RUL stimulation and brief (1 ms) during BT stimulation.

All patients received propofol (1–2 mg/kg) plus muscle relaxant succinylcholine chloride (0.3–0.8 mg.kg) and were randomly allocated to receive either ketamine (PANPHARMA, 0.5 mg/kg) or placebo (NaCl 0.9% in the same volume) injected intravenously before the injection of propofol. Anesthetists, the ECT team and psychiatrist raters were blind to the treatment conditions, and the pharmacist prepared the dose of ketamine or NaCl in a blind vial for the day of ECT. Associated pharmacological treatments remained stable throughout the study period.

### Statistical Analysis

As a primary analysis, we compared the number of patients who achieved remission (MADRS <10 at week 4, after eight ECT sessions) between groups by analyzing the proportions (Chi-square analysis) using JASP (Version 0.9.2) [JASP Team (2018) Computer software].

For secondary objectives, the number of patients who achieved remission throughout the study was compared between groups using Kaplan-Meier survival analysis throughout the study period in RStudio (R version 3.4.3). The number of patients who achieved remission at the endpoint (i.e., after a maximum of 16 ECT sessions) was compared between groups in the same manner as after eight ECT sessions (week 4). The changes in MADRS scores throughout the study (baseline, week 4, endpoint) within and between the two groups were analyzed using repeated measures ANOVA. The duration of seizures (clinical and EEG) and the severity of depressive symptoms (MADRS score) were compared between groups using Student’s t tests. As a secondary outcome analysis, we also compared the number of responders to ECT between groups at week 4 and the endpoint. Response was defined as a decrease of at least 50% in the MADRS score compared to baseline.

Statistical analyses were conducted using a strict intention-to-treat (ITT) principle. The analysis was conducted in a last-observation carried forward (LOCF) manner through the indicated time points. Patients without any changes in MADRS, patients who achieved remission before the 8^th^ session and patients who needed to be switched to another anesthetic agent (etomidate) or to another associated pharmacological treatment for clinical or safety reasons were excluded from the study (drop out), and corresponding data were analyzed with the LOCF method in the ITT sample until the endpoint. Clinical data from patients who continued ECT but with another anesthetic and from patients excluded for other reasons were not available.

## Results

There was no significant difference in the sociodemographic and clinical characteristics at baseline between groups (**Table 1**).

**Table 1:** Clinical and sociodemographic characteristics of patients with major depressive disorder.

	placebo + propofol	ketamine + propofol	p
n	16	11	
Age (years)	59.60 (15.71)	57.34 (13.02)	0.69
Gender M/F	9/7	7/4	0.70
Unipolar/bipolar depression (n)	13/3	11/0	0.25
Treatment resistant depression (n)	13	11	0.25
MADRS score	35.44 (4.77)	36.73 (8.81)	0.62
MMSE score	27.4 (2.4)	26.7 (4.7)	0.66
Prior suicide attempts (n)	0.5 (0.9)	1.5 (1.4)	0.07
ECT parameters			
Seizure threshold (mC)	119.7 (109.8)	94.40 (62.15)	0.51
Number of sessions	9.62 (4.51)	10.18 (4.42)	0.75
EEG seizure duration (s)	29.42 (12.66)	20.12 (6.82)	0.036*
Clinical seizure duration (s)	19.95 (9.74)	12.54 (3.78)	0.025*
RUL/BT	6/10	5/6	0.68

The results are given as the mean (standard deviation).

RUL, right unilateral; BT, bitemporal.

*means statistically significant.

### Primary Outcome, Number of Remitters at Week 4 (After Eight ECT Sessions)

**Figure 2:**
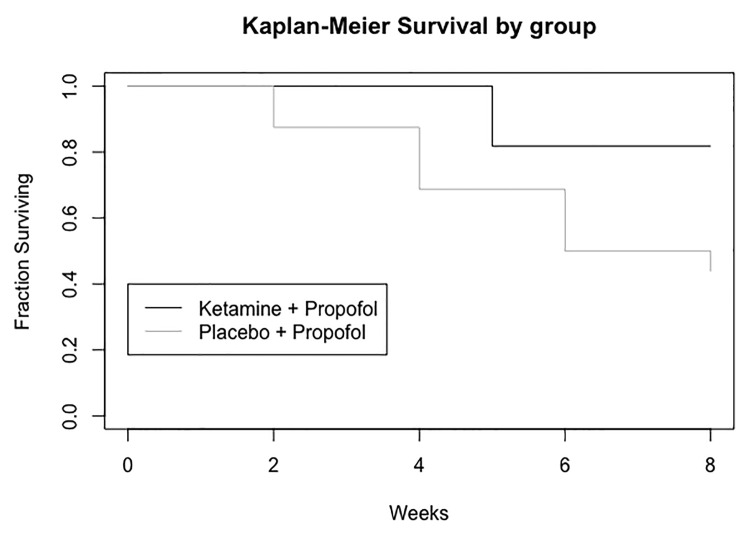
Number of patients who achieved remission (MADRS score < 10) throughout the study period while receiving placebo + propofol or ketamine + propofol during electroconvulsive therapy (ECT).

We compared the number of remitters at week 4 (after eight ECT sessions) between patients who received ketamine + propofol and those who received placebo + propofol (**Figure 2**). There were significantly fewer patients who achieved remission (remitters) in the ketamine + propofol group (0/11; 0%) than in the placebo + propofol group (5/16; 31%; χ^2^ = 4.22; p = 0.040).

Before the 8^th^ session of ECT, two patients were already remitters in the placebo group (one after four ECT sessions, one after six ECT sessions) and 0 remitters in the ketamine group (see **Figure 2**). Moreover, three patients from the placebo group and two patients from the ketamine group were excluded from the study before the 8^th^ session for clinical or safety reasons.

### Secondary Objectives

For clinical or safety reasons, one more patient from the ketamine group was excluded from the study after the 8^th^ session of ECT. At the end point (after a maximum of 16 ECT sessions), there was a significant difference in the total number of dropouts between the placebo group (four patients) and the ketamine group (7 patients) (p = 0.013). Finally, a large majority of patients did not receive the all 16 planned ECT sessions, and only the data collected before their exclusion were taken into account in the analysis.

Regarding the number of patients who achieved remission throughout the study period, the Kaplan-Meier analysis of survival rates revealed a trend toward a significant difference between the two groups, Chi² = 3.559, df = 1; p = 0.058, **Figure 2**.

At the endpoint (i.e., after a maximum of 16 sessions), there were significantly fewer remitters in the ketamine + propofol group (2/11; 18%) than in the placebo + propofol group (9/16; 56%; χ^2^ = 3.91; p = 0.048).

Regarding changes in MADRS scores throughout the study period, repeated measures ANOVA revealed no interaction between time and group [F_(2,48)_ = 1.507; p = 0.232; η²_p_ = 0.06], a significant effect of time [F_(2,48)_ = 45.463; p < 0.001; η²_p_ = 0.65] and no effect of group [F_(1,24)_ = 1.797; p = 0.193; η²_p_ = 0.07]. MADRS scores at the endpoint (after a maximum of 16 ECT sessions) were significantly different (Student’s t test; p = 0.04) between the ketamine + propofol group (22.7 ± standard deviation 13.6) and the placebo + propofol group (mean 13.0 ± 9.53). No difference was observed between groups at week 4 after eight ECT sessions (26.1 ± 11.5 in the ketamine + propofol group versus 19.1 ± 11.9 in the placebo + propofol group; p = 0.14). We also undertook a per protocol analysis at week 4 using repeated measures ANOVA. This analysis concerned 11 patients from the placebo group (the two remitters before the 8^th^ sessions were not included) and 9 from the ketamine group. No significant Group X Time interaction was observed (F_(1,18)_ = 1.533; p = 0.23). There was a significant effect of the time (F_(1,18)_ = 46.983, p <0.001) and no significant effect of the group (F_(1,18)_ = 0.122, p = 0.73). In the per protocol analysis at Week 4, three patients were remitters in the placebo group (+2 before the 8^th^ session), 0 in the ketamine group (ns).

Regarding analyses of the response rate (defined as a decrease of at least 50% in the MADRS score) after eight sessions of ECT, there was a significantly smaller number of patients who were qualified as responders in the ketamine + propofol group (0/11; 0%) than in the placebo + propofol group (5/16; 31%; χ^2^ = 4.22; p = 0.040). The difference was not significant at the endpoint (4/11 versus 11/16; χ^2^ = 2.77; p = 0.096).

We observed a significant difference in the duration of the seizures between groups. The durations of clinical and EEG seizures were significantly shorter in the ketamine group than in the placebo group (**Table 1**). However, there was no correlation between clinical improvement and the duration of EEG seizure duration (r = −0.081; p = 0.687).

## Discussion

The aim of this study was to investigate whether the combination of ketamine and propofol would accelerate remission in patients with severe MDE who underwent eight ECT sessions and would increase the remission rate after 16 sessions compared with patients who received placebo + propofol. Strikingly, in contrast to our hypothesis, we observed a smaller number of patients who were remitters (MADRS < 10) in the ketamine group than in the placebo group at week 4, after a maximum of eight ECT sessions. Moreover, no significant differences in the number of remitters by week or in the changes in the MADRS score were observed between groups throughout the study period. However, the results at the endpoint (after a maximum of 16 ECT sessions) should be interpreted cautiously since the majority of patients did not receive the 16 planned sessions; an average of 9.62 (4.51) sessions were delivered in the placebo group and 10.18 (4.42) in the ketamine group. Moreover, compared to the placebo arm, a significantly larger proportion of patients who received ketamine were prematurely excluded from the study for clinical or safety reasons during the blind phase of the study. Nevertheless, these results are in line with previous studies that did not find any beneficial effect of using ketamine among patients undergoing ECT (Abdallah et al., 2012; Anderson et al., 2017; Fernie et al., 2017; Zhang et al., 2018; Shams Alizadeh et al., 2015). The current study therefore supports the recommendation of not using the combination of ketamine + propofol in the ECT setting since we observed a significantly smaller number of remitters in the ketamine + propofol group than in the placebo + propofol group after eight ECT sessions no difference throughout the study period and a higher number of exclusions from the study for clinical and safety reasons in the ketamine arm.

Ketamine and propofol are both widely used anesthetic agents that are assumed to operate *via* two distinct mechanisms of action. Ketamine is a noncompetitive antagonist of *N*-methyl-d-aspartate (NMDA) receptors, whereas propofol potentiates GABA_A_-gated receptor currents. Although it has been established that propofol can inhibit or eliminate some adverse effects of ketamine in many clinical conditions and procedures (Sinner and Graf, 2008), the combination of them can lead to surprising results. For instance, EEG studies have reported that while ketamine alone results in a downshift of the alpha peak (an indicator of the quality of anesthesia) and propofol keeps it roughly constant (Bojak et al., 2013), the combination of these drugs shift of the alpha peak to higher frequency by up to 4 Hz. These results suggest that the effect of ketamine can be markedly altered in the presence of propofol (Tsuda et al., 2007). Here, we observed that ketamine might also interact with propofol to decrease the remission rate in patients who received ECT.

We also observed that the duration of seizures was shorter in patients who received ketamine + propofol than in the placebo + propofol group. This effect on the duration of seizures was unexpected and contrasts with previous literature (Zhong et al., 2016; Zhang et al., 2018). Although the relationship between seizure length and clinical outcome with ECT remains unclear (Fear et al., 1994; Mårtensson et al., 1994), the shorter seizure length in the ketamine + propofol group could explain the smaller number of remitters and the higher number of dropouts (switched to etomidate) observed in this group. This decrease in the duration of seizure could also be taken into account to explain the higher number of switches to etomidate observed in patients from ketamine + propofol in the current study. However, this should be tempered because no correlation between seizure duration and changes in MADRS scores was observed in the current study. The anesthetic-ECT time interval, the overall dose of anesthetic and the depth of anesthesia may also have an effect on the clinical outcomes with ECT (Gálvez et al., 2016; Asztalos et al., 2018). In the current study, ketamine (or placebo) was injected as a slow intravenous bolus before propofol (also injected as a slow intravenous bolus), and ECT stimulation was delivered several minutes after propofol injection in both groups. The anesthetic-ECT time interval was therefore different between the two groups, suggesting that the ECT stimulation was not delivered during an optimal blood level of anesthetic concentration in the ketamine + propofol group (Gálvez et al., 2016). Further studies are required to determine the order and the optimal anesthetic-ECT time interval after the injection of the combination of ketamine and propofol.

These results cannot be generalizable to all indications of ECT. Our sample of patients was a mixed sample of aging patients (mean age 56 years old) with severe depression (MADRS scores ranging from 20 to 50, mean 36.1 ± 6.6): some patients reported previous suicide attempts (range 0–5, mean 0.9 ± 1.2); we had patients of both genders (16 males, 11 females); and we had patients with uni- and bipolar depression. Several studies have reported that ketamine alone could have different effects on depression (Rong et al., 2018) depending on gender (Coyle and Laws, 2015, but see also Freeman et al., 2019), the type of depression (uni- or bipolar; Thomas et al., 2018), prior suicide attempts, family history of alcohol use disorder or body mass index (Niciu et al., 2014; Rong et al., 2018). However, the size of our sample did not allow a specific investigation of the weight of all these factors in our study. As an example, only three patients with bipolar depression were included in the current study. All of them were randomly allocated to the placebo condition, and they were all remitters at the end of the study period. A large majority of patients presented with treatment-resistant depression. Only three patients from our sample were referred to ECT treatment because of the severity of unipolar MDE without meeting the criteria of treatment-resistant depression. They were randomly allocated to the placebo condition, and at the end of the study period, two were nonremitters. Further studies are needed to investigate the role of these factors on the response to ECT and ketamine. Moreover, we observed a trend toward a significant difference between groups regarding the number of prior suicide attempts that may have influenced clinical outcomes as well as the number of dropouts between groups and the use of either RUL or BT stimulation. We also have no control on the level of psychotic symptoms in the sample. Our primary outcome was chosen after eight sessions of ECT, at half regimen regarding the total of 16 planned ECT. However since a majority of patients did not received the full regimen of ECT, our time point of measure seems not optimal to assess early response to ECT. Indeed, in a large study including 253 patients with depression, it has been reported that 65% of patients were already remitters at or before the 10^th^ session of ECT (Husain et al., 2004). However, it is also important to note that we used a titration method in the current study, and thus, the first session (as well as the 2^nd^ session in some cases) was not an effective ECT session with a significant seizure. Nevertheless, further large studies specifically design to investigate this point and assessing remission rate after two or three effective ECT sessions are required to determine the real short term advantage of ketamine even if no early effect was observed in the current study.

Although some studies have highlighted a procognitive effect of ketamine given alone, in the current study, we have no measures of the effects of the combination of ketamine + propofol on cognitive abilities or on cognitive complaints of patients. However, since no superior clinical effects were observed in the ketamine + propofol group, there is no interest in investigating the cognitive effects of this combination in further studies.

The results from the current study corroborated previous studies from the literature and did not support the use of the combination of ketamine and propofol as anesthetic agents for ECT in patients with major depression in clinical settings. However, further studies are needed to investigate the beneficial clinical and cognitive effects of ketamine alone in ECT settings and to determine the optimal dose and duration to administer and targeted population.

## Data Availability Statement

The raw data supporting the conclusions of this article will be made available by the authors, without undue reservation.

## Ethics Statement

The studies involving human participants were reviewed and approved by CPP sud Est 6, France. The patients/participants provided their written informed consent to participate in this study.

## Author Contributions

JB, EP, FG, SI, and M-FS-C designed the study. SI, EP, FG, MP, LS, and RG included participants and rated clinical scales. SI, JB, and LS collected the raw data, LS and JB undertook analysis and wrote the first draft of the manuscript. All authors contributed to the article and approved the submitted version.

## Funding

This work was sponsored by academic research (CH Le Vinatier Scientific council; grant number CSR B11). The funders had no role in the design, analysis, write-up or decision to submit for publication.

## Conflict of Interest

The authors declare that the research was conducted in the absence of any commercial or financial relationships that could be construed as a potential conflict of interest.
